# Supplementary material for: Single-molecule visualization of conformational changes and substrate transport in the vitamin B12 ABC importer BtuCD-F
Source: Nat Commun. 2017 Nov 21;8:1652. doi: 10.1038/s41467-017-01815-7 (PMC5698293; doi:10.1038/s41467-017-01815-7)
Supplement: Supplementary file 1 — Supplementary Information [file 41467_2017_1815_MOESM1_ESM.pdf]

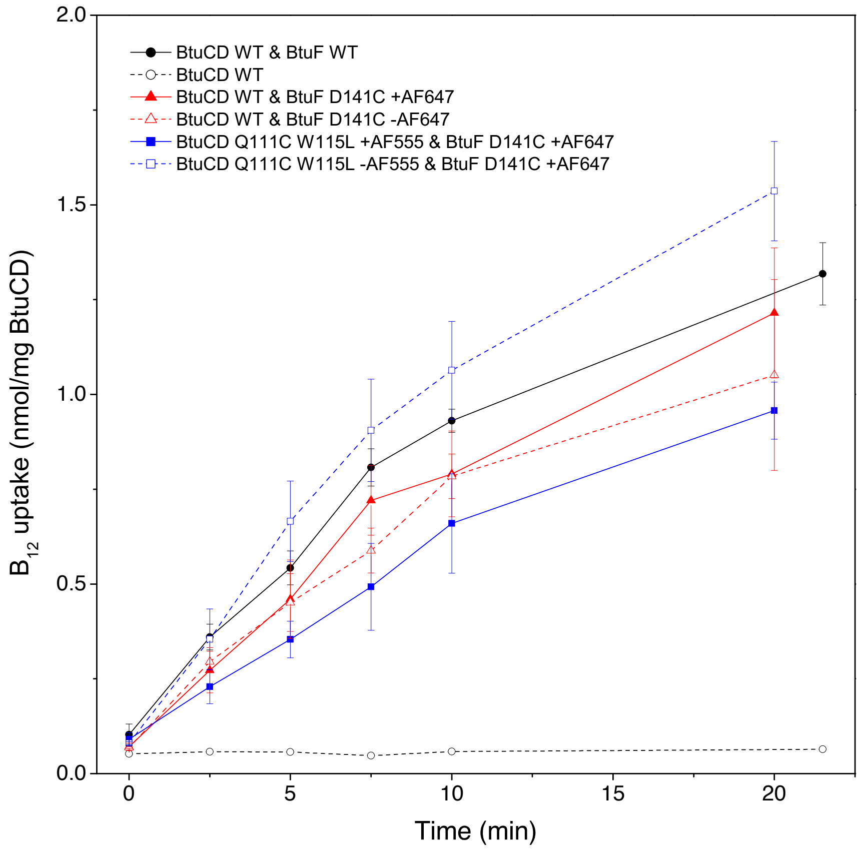

Supplementary figure 1 – Radiolabelled substrate transport by reconstituted BtuCD-F

Uptake of [^{57}Co]vitamin B_{12} in proteoliposomes. BtuCD and BtuF were purified and labelled as described before (see Methods), and reconstituted into proteoliposomes as described by Borths *et al.* [1]. Uptake of [^{57}Co]vitamin B_{12} was measured essentially as described by Borths *et al.* [1], but with the omission of an ATP regenerating system. Liposomes were loaded (see Methods) with 2 mM ATP and 10 mM $MgCl_2$, extruded at 400 nm and washed by means of centrifugation and resuspension. Reactions mixture contained ~3 mg ml^{-1} lipids and ~0.3 μM BtuCD and reactions were initiated by adding 1.0 μM BtuF and 0.1 μM of [^{57}Co]vitamin B_{12} on the outside. Each measurement was performed in triplicate; mean and standard deviation are plotted. Measurements were performed in sets of two (labelled by same line colour and symbol shape) from the same batch of reconstitution. Uptake rates should not be considered as absolute numbers, because accumulated ADP might inhibit the transporter, there is a possibility of multi-lamellar 400-nm proteoliposomes and during the filtering step in the method sample is lost. However, measured rates agree well with data reported by Borths *et al.* [1].

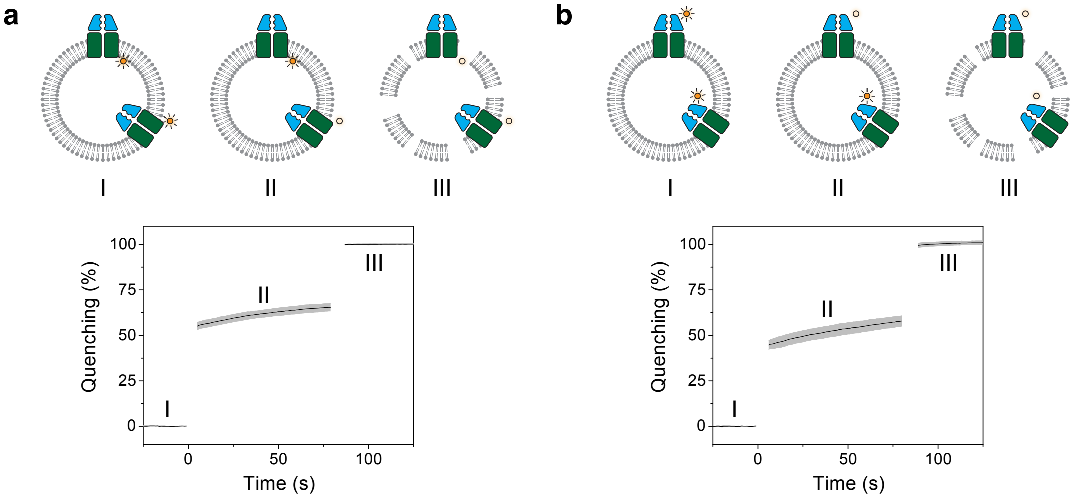

Supplementary figure 2 – Orientation of BtuCD in liposomes by fluorescein quenching

The orientation of membrane proteins can be determined by site specific labelling of the protein with fluorescein and fluorescence quenching of the dye at low pH. To this end, two mutants of BtuCD are used: $BtuCD_{cys}$, with the cysteine labelling site on the periplasmic side, and $BtuCD_{D-cys}$, a newly designed mutant with a cysteine added to the N-terminus of BtuD and thus a labelling position on the cytoplasmic side. BtuCD was reconstituted with a mass ratio of BtuCD:lipids of 5:800, subjected to 4 freeze-thaw cycles, extruded at 100 nm and washed twice by means of centrifugation and resuspension in buffer (50 mM KP_i, 200 mM KCl). Initial reaction mixtures contained ~0.2 µM BtuCD in 1000 µl.

(a) $BtuCD_{cys}$. Fluorescein fluorescence was continuously measured (excitation/emission: 493/515 nm). Initially, fluorescence is maximum (and by definition no quenching occurs, I). Upon addition of 200 µl of 250 mM citric buffer pH 4.0 to the outside of the liposomes at time zero, fluorescence of right-side-out orientated proteins is quenched by lowering of the pH (II). Leaking of protons into the proteoliposomes causes a gradual increase in quenching. Finally, around 80 seconds, the fluorescence quenching reaches a maximum as 20 µl of 10% Triton X-100 is added to permeabilize the liposomes (III). The curve is normalized to the final value and error bands (standard deviation of four curves) are indicated by the grey area. The fraction of right-side-out orientated proteins can be inferred from the amount of quenching around 1 second: 55%. (b) Complementary experiment to (a): $BtuCD_{D-cys}$ is used instead of $BtuCD_{cys}$. Similarly, the fraction of inside-out oriented proteins is deduced from the amount of quenching around 1 second: 45%.

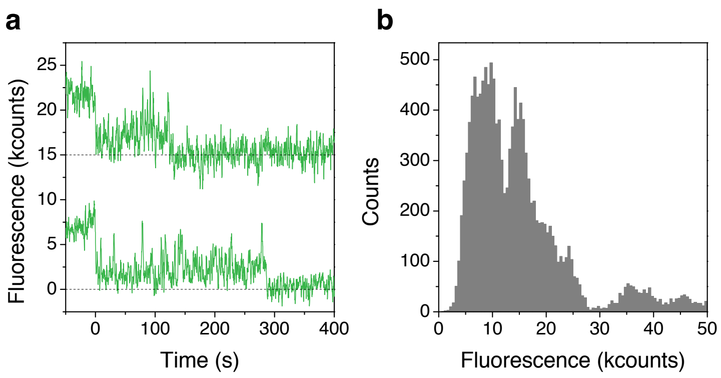

Supplementary figure 3 – Single BtuCD molecules in liposomes

(a) A complex of $BtuCD_{cys}$ labelled with Alexa Fluor 555 and unlabelled BtuF showed decrease in fluorescence intensity upon addition of ATP and Mg^{2+} at 0 seconds (**Fig. 1c**). Two example traces with bleaching after 130 and 280 seconds respectively are displayed here, indicating these signals originate from a single fluorophore on a single BtuCD molecule. (b) Plotting the distribution of the fluorescence intensity one minute before the introduction of ATP of all traces with an event show that a single fluorophore has an intensity between roughly 5000 and 12 000 counts.

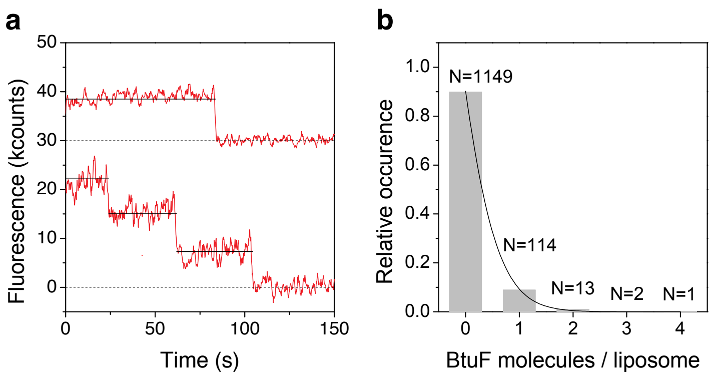

Supplementary figure 4 – Distribution of single BtuF molecules inside liposomes

Empty liposomes (stained with DiI, DiI:lipid molar ratio 1:25 000) were loaded with 0.32 µM BtuF (100% labelled with Alexa Fluor 647) and extruded at 100 nm, resulting in, on average, a theoretical value of 0.08 BtuF molecules per liposome. (a) Single liposomes, immobilised on glass and imaged in TIRF, were identified by their lipid markers. The corresponding number of BtuF molecules was determined by analysing bleaching steps. Two example traces with 1 and 3 bleaching steps are shown in panel (a); fluorescence levels are marked with a black line. (b) The distribution of BtuF molecules per liposome was plotted in a histogram. By fitting a Poisson distribution an average value of 0.10 +/- 0.01 BtuF molecules per liposome was found, which is in agreement with the expected value of 0.08.

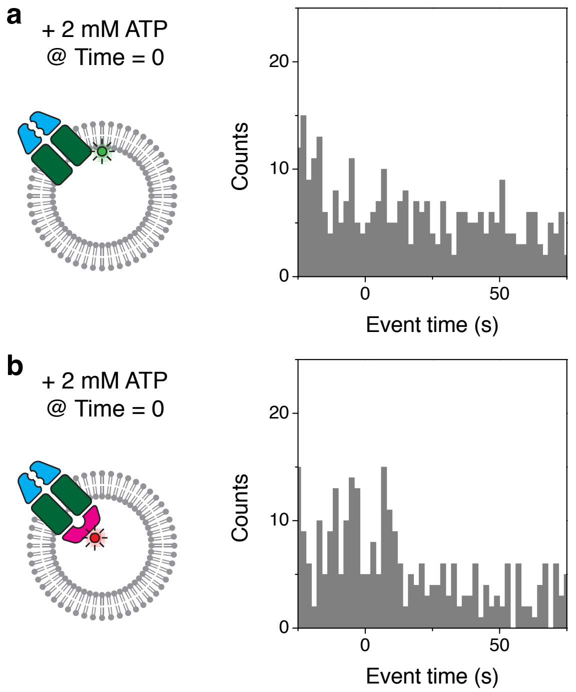

Supplementary figure 5 – Control experiments

(a) Liposomes reconstituted with $BtuCD_{cys}$ (labelled with Alexa Fluor 555), but devoid of BtuF, do not show any change in fluorescence intensity upon addition of ATP and Mg^{2+}. Only false events caused by noise are registered and no clear peak around 0 seconds is visible in the histogram – as opposed to the case when BtuF is present inside the liposomes (**Fig. 1d**). (b) When proteoliposomes with unlabelled BtuCD are loaded with BtuF (labelled with Alexa Fluor 647) no true positive events, that should emerge as a clear peak in the event histogram when ATP is introduced around 0 seconds, are registered.

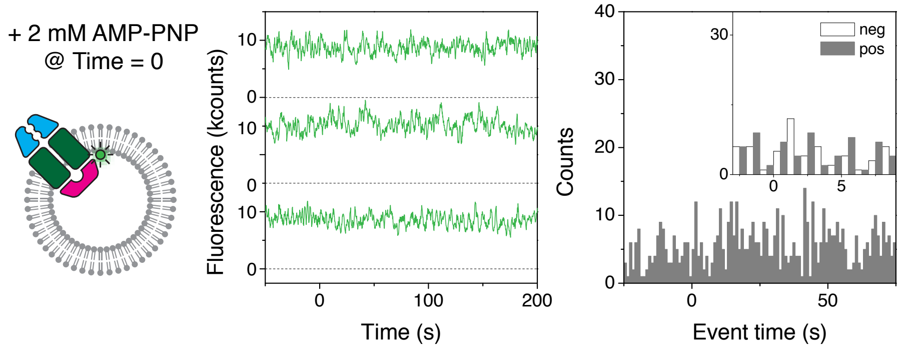

Supplementary figure 6 – AMP-PNP

A complex of $BtuCD_{cys}$ labelled with Alexa Fluor 555 and unlabelled BtuF does not decrease in fluorescence intensity upon addition of 2 mM AMP-PNP and 10 mM Mg^{2+} on the outside (middle panel). The distribution of event times of the first drop of intensity is plotted in a histogram (right panel) for the positive (pos, with AMP-PNP) and negative (neg, without AMP-PNP) experiment. Data are similar to data shown in **Fig. 1e**.

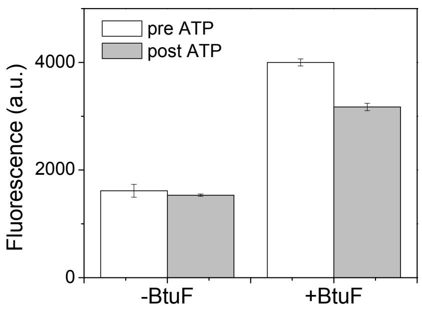

Supplementary figure 7 – Effect of BtuF and ATP on labelled BtuCD in bulk

$BtuCD_{cys}$ labelled with Alexa Fluor 555 was reconstituted (on average 0.5 BtuCD molecules per liposome) with or without unlabelled BtuF (if applicable, 2 μM) in 100-nm liposomes. Bulk fluorescence intensity was measured with a plate reader before and after addition of an ATP-Mg^{2+} mixture (2 mM ATP and 10 mM $MgCl_2$); data were corrected for dilution and time effects. When BtuF is present, BtuCD and BtuF interact and as a result the fluorescence of the Alexa Fluor 555 label on BtuCD increased. Upon addition of ATP, the fluorescence intensity of BtuF loaded proteoliposomes decreased, caused by the destabilization of the interaction between both proteins. The fluorescence intensity does not decrease to the level of fluorescence intensity of the proteoliposomes devoid of BtuF, since ATP can only interact with a fraction of the BtuCD proteins due to their orientation.

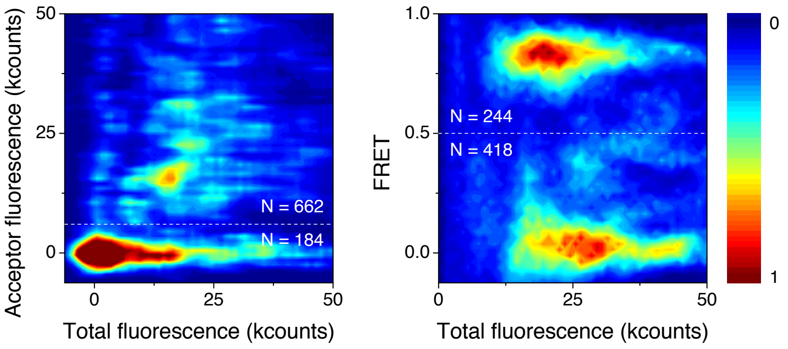

Supplementary figure 8 – FRET distribution of BtuCD-F complexes

A stable complex of $BtuCD_{cys}$ (Alexa Fluor 555) and BtuF (Alexa Fluor 647) was formed when reconstituted in substrate-free liposomes (**Fig. 3a**). The left panel shows a two-dimensional histogram of the relative occurrence of acceptor fluorescence (acceptor excitation) versus total fluorescence (donor excitation, sum of donor and acceptor fluorescence). Liposomes with at least one fluorescent BtuF molecule (acceptor fluorescence above 6000 counts; indicated by the dashed line) in the lumen are further analysed (right panel). Here, the FRET value is plotted versus the total fluorescence; 244 out of 662 proteoliposomes show a high FRET value of on average 0.83, indicative of complex formation. A 2.5x higher laser power density was used in these experiments.

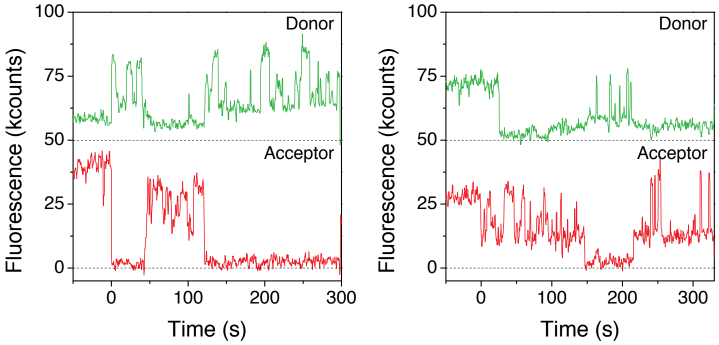

Supplementary figure 9 – Rare example traces that show exchange of BtuF

The probability of finding two or more copies of BtuF inside a liposome is roughly 25% when the average copy number is one. With the labelling efficiencies of BtuF ranging from 70% to 90%, in approximately 5% to 10% of the liposomes a pair of labelled and unlabelled BtuF is present. In rare cases, many times less than expected from the indicated probabilities, an exchange could be observed between labelled and unlabelled BtuF (see plots). Only after introduction of ATP at 0 seconds, dynamics – this is no blinking as explained in the main text – are present. It is also observed that the dynamics originate from the donor dye **(Fig. 1)** and are transferred to the acceptor dye if present in the form of bound labelled BtuF. Note: the right figure shows a green trace that is convoluted with the fluorescence of a nearby liposome until approximately 25 seconds.

Supplementary table 1 – Theoretical effect of vitamin B_{12} quenching on fluorophores

**Fluorophore**	**Förster radius (R_0)**	**Quenching (E) when vitamin B_{12} is bound to labelled BtuF**
Alexa Fluor 488	45.9 Å	84 %
Alexa Fluor 555	29.1 Å	25 %
Alexa Fluor 647	20.4 Å	4 %

Vitamin B_{12}, which has a high extinction coefficient, quenches fluorescence of nearby dyes based on FRET (vitamin B_{12} is the acceptor, but does not emit fluorescence). The amount of quenching equals the FRET efficiency E, which is defined as $E = \frac{1}{1+\left(r/R_0\right)^6}$. Here, r is the distance between donor and acceptor and R_0 the Förster radius. For vitamin B_{12} bound to labelled BtuF $r \approx$ 35 Å. The Förster radius in units of Å can be calculated as $R_0 = 0.2108\left(\kappa^2 \Phi_0 n^{-4} J\right)^{1/6}$. In this equation, κ^2 is the orientation factor (estimated at 2/3), Φ_0 the quantum yield of the dye and n refractive index of the medium (water: 1.33). J is the spectral overlap integral in units of $M^{-1}cm^{-1}nm^4$: $J = \int_0^\infty F_D(\lambda)\varepsilon_A(\lambda)\lambda^4\, d\lambda$, where F_D is the donor emission spectrum (its integral normalised to one), ε_A the acceptor absorption spectrum (with its maximum scaled to the molar extinction coefficient in units of $M^{-1}cm^{-1}$) and λ the wavelength in units of nm. The resulting Förster radii and quenching values for several dye – vitamin B_{12} pairs are displayed in the table.

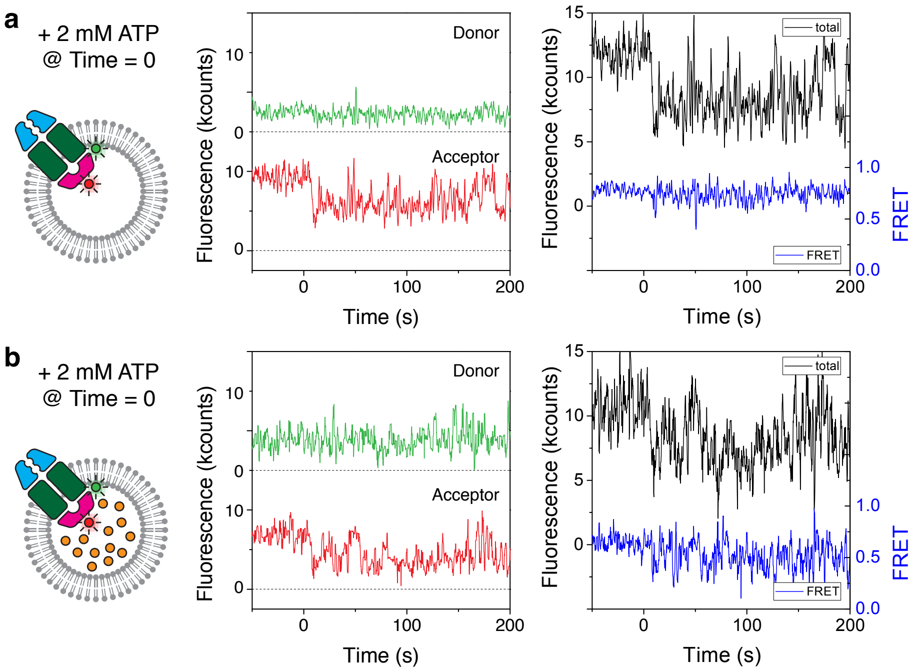

Supplementary figure 10 – Complex formation observed with FRET – total intensity and FRET

Complementary to **Fig. 2**. For both (a) and (b), data in the left panels is equal to **Fig. 2b and c**. The right panels show total intensity (black, sum of donor and acceptor) and FRET (blue, acceptor divided by total intensity) traces.

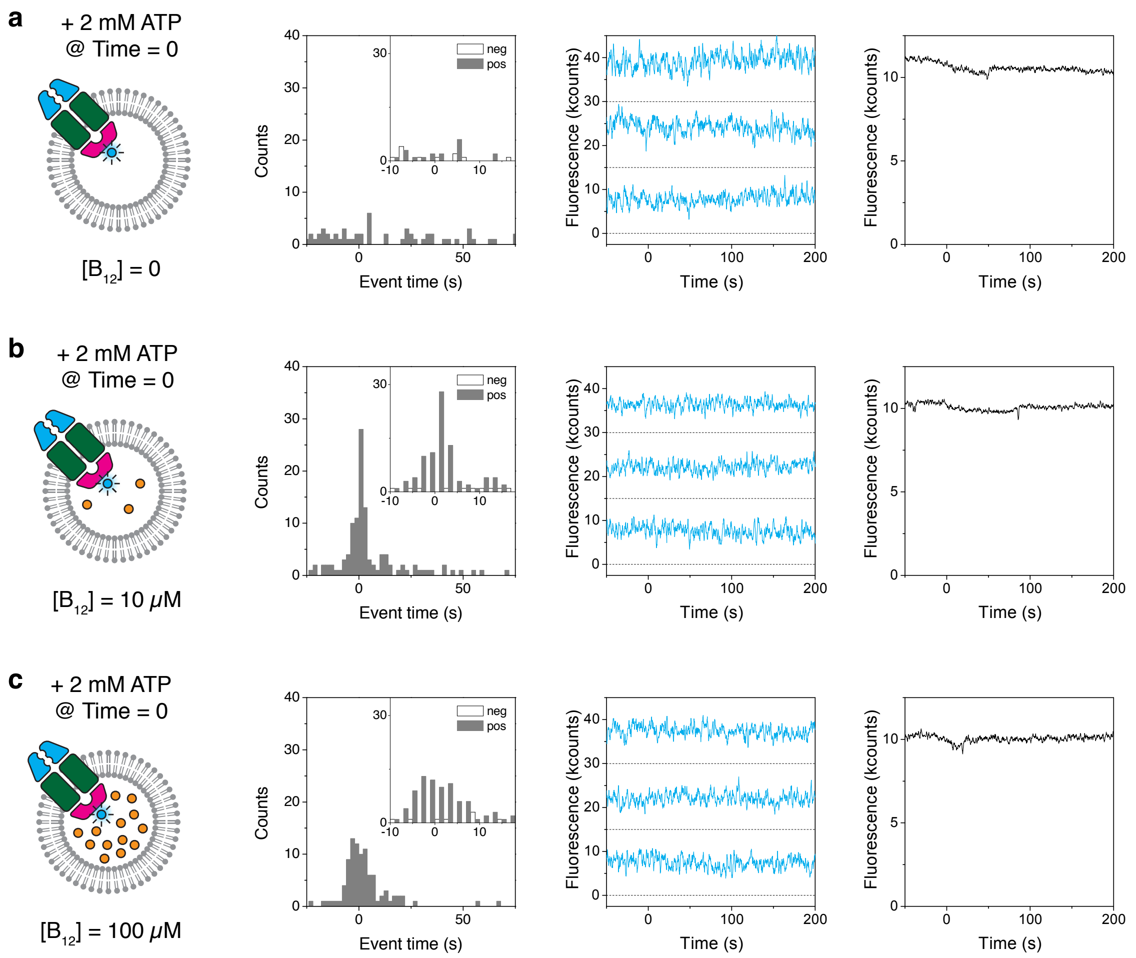

Supplementary figure 11 – Distribution of events of vitamin B_{12} quenching on reconstituted BtuCD-F

Upon addition of ATP to vitamin B_{12}-loaded liposomes, the fluorescence signal is quenched **(Fig. 4)**. The distributions of event times (times at which the fluorescence intensity decreases) are plotted in the histograms. Only when vitamin B_{12} was loaded into the lumen, events were detected, as the histograms show (panels (b) and (c), versus no events in panel (a)). The control experiment (neg) differs from the positive experiment (pos) by the omission of ATP and Mg^{2+} from the activation buffer.

Traces that do not show an event with a decrease in fluorescence intensity, also lack events with an increase in fluorescence intensity at any time, and thus remain flat (middle column: individual traces, right column: sum of all these traces). Fluctuations in the summed signals are due to experimental variations.

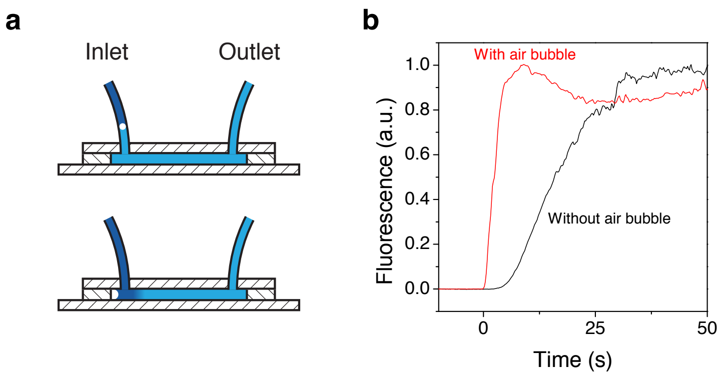

Supplementary figure 12 – Flow-cell design for rapid buffer exchange

(a) Flow cells were constructed as described previously [2], with the addition of a cavity at the position where the inlet tube enters the channel. A tiny air bubble in the inlet tube physically separates the two buffers, preventing the mixing of the buffers before entering the flow cell. When the air bubble enters the channel, it becomes trapped in the cavity and the two buffers form one continuous phase only moments before arriving at the point of observation at the centre of the channel. (b) Only inside the channel the interface starts to diffuse, thus giving rise to a sharp buffer transition at the measurement point, as opposed to the case in which no air bubble is present in the inlet tube to partition the buffers and diffusion already takes place in the inlet tube. Both curves are measured in TIRF by introducing rhodamine B and measuring the total fluorescence intensity of non-specifically surface-bound dye molecules. With the typical flow speeds used in our experiments, 80% increase is reached in two seconds. Increasing the flow speed improves the sharpness of the transition, but also introduces focal drift in our current setup. With the new setup, a drop in intensity is observed after a few seconds, which could be caused by non-laminar flow effects.

Supplementary table 2 – Primers for mutations in BtuCD-F

Mutation	Primer
BtuC Q111C	forward:<br>5’-GTATTGCTTGGGCAAGGGTGCCTCCCCAACTGGGCGCTA-3’
	reverse:<br>5’-TAGCGCCCAGTTGGGGAGGCACCCTTGCCCAAGCAATAC-3’
BtuC W115L	forward:<br>5’-CAAGGGTGCCTCCCCAACCTGGCGCTAGGGCTGAGCGCG-3’
	reverse:<br>5’-CGCGCTCAGCCCTAGCGCCAGGTTGGGGAGGCACCCTTG-3’
BtuD E159Q	forward:<br>5’-CAATTGCTGCTTCTTGATCAGCCGATGAACAGTCTTGAT-3’
	reverse:<br>5’-ATCAAGACTGTTCATCGGCTGATCAAGAAGCAGCAATTG-3’
BtuF D141C	forward:<br>5’- GCCGCGCAATCCCTGCTGTGCCAGTACGCGCAATTGAAA-3’
	reverse:<br>5’- TTTCAATTGCGCGTACTGGCACAGCAGGGATTGCGCGGC-3’

Supplementary references

1 Borths, E. L., Poolman, B., Hvorup, R. N., Locher, K. P. & Rees, D. C. In vitro functional characterization of BtuCD-F, the Escherichia coli ABC transporter for vitamin B12 uptake. *Biochemistry* **44**, 16301-16309, doi:10.1021/bi0513103 (2005).

2 Tanner, N. A. & van Oijen, A. M. Visualizing DNA replication at the single-molecule level. *Methods Enzymol* **475**, 259-278, doi:10.1016/S0076-6879(10)75011-4 (2010).
